# Supplementary material for: Disease Differentiation and Monitoring of Anti-TNF Treatment in Rheumatoid Arthritis and Spondyloarthropathies
Source: Int J Mol Sci. 2021 Jul 9;22(14):7389. doi: 10.3390/ijms22147389 (PMC8307996; doi:10.3390/ijms22147389)
Supplement: Supplementary file 1 [file ijms-22-07389-s001.zip › Supp.pdf]

Disease differentiation and monitoring of anti-TNF treatment in rheumatoid arthritis and spondyloarthropathies

Katarzyna Bogunia-Kubik[1], Wojciech Wojtowicz[2], Jerzy Swierkot[3], Karolina Anna Mielko[2#], Badr Qasem [2#], Joanna Wielinska[1], Renata Sokolik[3], Łukasz Pruss[4], Piotr Młynarz[2*]

[1] Laboratory of Clinical Immunogenetics and Pharmacogenetics, Hirszfeld Institute of Immunology and Experimental Therapy, Polish Academy of Sciences, Wroclaw, Poland; katarzyna.bogunia-kubik@hirszfeld.pl (K.B.K); joanna.wielinska@hirszfeld.pl (J.W);

[2] Department of Rheumatology and Internal Medicine, Wroclaw Medical University, Wroclaw, Poland; jerzy.swierkow@umed.wroc.pl (J.S); renata.sokolik@umed.wroc.pl (R.S);

[3] Department of Bioorganic Chemistry, Wroclaw University of Technology, Wroclaw, Poland; piotr.mlynarz@pwr.edu.pl (P.M); wojciech.wojtowicz@pwr.edu.pl (W.W); badr.qasem@pwr.edu.pl (B.Q); karolina.mielko@pwr.edu.pl (K.M)

[4] Ardigen, Krakow, Poland; lukasz.pruss.1993@gmail.com (L.P)

* Correspondence: piotr.mlynarz@pwr.edu.pl (P.M); (ORCID: 0000-0002-8502-4022)

Supplemental material

Table S1. Demographic data and clinical profiles of patients treated with TNF inhibitors.

**Patients**	**RA**	**AS**	**PsA**
**Number of patients;** n	26	29	23 (20 peripheral + 3 axial)
Age, median (range); years	55 (23-74)	45 (26-75)	43 (29-71)
**Sex**; females/males	21/5	7/22	9/14
**Disease duration**, median (range); years	9 (1-56)	11 (1-40)	8 (2-41)
**Disease onset**, median (range); years	43 (4-64)	31 (12-45)	35 (18-56)
**Anti-CCP positive**, n (%)	18 (69%)	-	-
**RF positive**, n (%)	20 (77%)	-	-
**HLA B27 positive**, n (%)	-	27 (93%)	-
**Concomitant treatment at the start of biologic treatment:**			
MTX, n (%)	17 (65%)	9 (31%)	15 (65%)
Corticosteroids, n (%)	14 (54%)	5 (17%)	2 (9%)
**Treatment (anti TNF drug):**			
Etanercept, n (%)	14 (54%)	11 (38%)	8 (35%)
Golimumab, n (%)	3 (11.5%)	2 (7%)	3 (13%)
Adalimumab, n (%)	3 (11.5%)	10 (35%)	10 (43%)
Certolizumab, n (%)	6 (23%)	5 (17%)	2 (9%)
Infliximab, n (%)	-	1 (3%)	-

**Disease activity:**			
DAS28 before treatment, mean ± SD	6.10 ± 0.62 (range: 5.12-7.19)	-	6.17 ± 0.94 (range: 4.4-8.3)
DAS28 at 6 months, mean ± SD	2.69 ± 0.84 (range: 0.84-4.93)	-	3.15 ± 1.44 (range: 1.01-6.06)
Remission at 6 months, n (%) (DAS28<2.6)	9 (35%)	-	8 (40% of peripheral PsA)
Low disease activity at 6 months, n (%) (2.6≤DAS28≤3.2)	13 (50%)	-	4 (20% of peripheral PsA)
BASDAI before treatment, median (range)	-	8.2 (5.7-10)	8 (6-9)
BASDAI at 6 months, median (range)	-	2.15 (0.75-9)	2 (0-8)
Remission at 6 months (BASDAI<3), n (%)	-	26 (90%)	3 (100% of 3 axial PsA)
Non-responder at 3 months, n (%)	7 (27%)	3 (10%)	9 (39%)
Non-responder at 6 months, n (%)	4 (15%)	2 (7%)	8 (35%)
Treatment efficacy after 3 months - responders (good and moderate response), n (%)	19 (73%)	26 (90%)	14 (61%)
Treatment efficacy after 6 months - responders (good and moderate response), n (%)	22 (85%)	27 (93%)	15 (65%)

RA= rheumatoid arthritis, AS= ankylosing spondylitis, PsA= psoriatic arthritis
DAS28 = disease activity score 28; CRP = C-reactive protein; RF = rheumatoid factor; anti-CCP = anti-cyclic citrullinated peptide antibodies; SD = standard deviation; TNF = tumor necrosis factor, MTX= methotrexate; NSAIDs= Nonsteroidal anti-inflammatory drugs

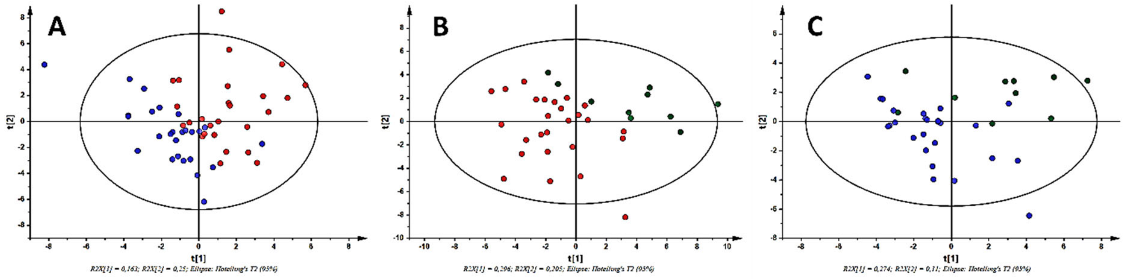

Figure S1. PLS-DA model plots for RA treatment at various time points. A – before treatment vs. after 3 months, B – before treatment vs. after 6 months, C – after 3 months vs. after 6 months of therapy with TNFi. Red – RA patients before treatment, Blue – 3 months after initialization of anti-TNF treatment, Green – 6 months after treatment with TNFi.

Table S2. The VIP score for discrimination PLS with univariate analysis results for quantified metabolites in comparisons for RA patients.

Rheumatoid arthritis												
**Metabolite**	**VIP score for PLS-DA model**			**Precentage diffrence [%]**			***p value***			**Adjusted *p value***		
	**BT vs 3M**	**BT vs 6M**	**3M vs 6M**	**BT vs 3M**	**BT vs 6M**	**3M vs 6M**	**BT vs 3M**	**BT vs 6M**	**3M vs 6M**	**BT vs 3M**	**BT vs 6M**	**3M vs 6M**
**2-Hydroxybutyrate**	0.923	1.142	0.523	-9.98	3.16	13.13	3.95E-01	9.03E-01	1.13E-01	6.27E-01	9.03E-01	4.77E-01
**2-Oxoisocaproate**	1.165	1.239	0.898	16.07	30.10	14.21	2.86E-02	8.24E-04	1.16E-01	1.36E-01	8.57E-03	4.77E-01
**3-Hydroxybutyrate**	0.574	0.958	0.263	-27.70	-50.73	-23.86	4.32E-01	6.05E-02	9.94E-01	6.27E-01	1.42E-01	9.94E-01
**3-Methyl-2-oxovalerate**	0.996	1.198	0.905	16.21	37.25	21.37	2.05E-01	2.46E-03	1.16E-01	4.63E-01	1.53E-02	4.77E-01
**Acetate**	0.969	0.879	1.427	-18.72	-30.55	-12.01	7.37E-02	4.79E-03	4.49E-02	2.56E-01	2.49E-02	4.67E-01
**Acetone**	0.640	0.917	0.652	1.43	9.81	8.38	4.34E-01	2.86E-01	2.26E-01	6.27E-01	3.91E-01	5.68E-01
**Alanine**	1.643	1.448	0.919	26.23	35.02	9.00	1.27E-05	2.58E-04	5.06E-01	3.31E-04	6.70E-03	6.80E-01
**Choline**	1.284	1.545	1.353	-14.61	8.29	22.84	1.18E-01	4.34E-01	1.19E-01	2.94E-01	5.25E-01	4.77E-01
**Citrate**	0.470	0.548	0.564	5.71	12.44	6.74	4.03E-01	4.21E-01	2.50E-01	6.27E-01	5.21E-01	5.68E-01
**Creatine**	0.530	0.887	1.287	1.05	32.69	31.67	5.27E-01	1.35E-02	7.37E-03	7.05E-01	5.41E-02	2.11E-01
**Creatinine**	0.708	1.148	0.903	3.01	11.52	8.51	2.62E-01	8.40E-02	2.70E-01	5.05E-01	1.62E-01	5.68E-01
**Ethanol**	2.603	1.980	1.097	-68.73	-80.27	-13.39	5.28E-07	1.00E-04	2.65E-01	2.75E-05	5.20E-03	5.68E-01
**Formate**	0.659	0.275	1.705	14.33	-2.32	-16.63	2.78E-01	8.89E-01	8.99E-02	5.16E-01	9.03E-01	4.77E-01
**Glucose**	0.503	0.557	0.296	7.72	4.19	-3.53	2.58E-01	4.79E-01	9.88E-01	5.05E-01	5.66E-01	9.94E-01
**Glutamate**	0.495	0.645	1.538	2.59	-6.14	-8.73	7.61E-01	5.69E-01	4.30E-01	8.25E-01	6.30E-01	6.57E-01
**Glutamine**	1.370	1.213	0.830	14.34	22.63	8.37	2.48E-03	2.65E-03	2.32E-01	4.29E-02	1.53E-02	5.68E-01
**Glycerol**	0.656	0.619	0.745	-6.90	-2.58	4.32	3.05E-01	4.16E-01	4.52E-01	5.47E-01	5.21E-01	6.71E-01
**Glycine**	0.835	0.807	0.748	10.16	8.72	-1.44	2.22E-02	7.38E-02	5.10E-01	1.33E-01	1.48E-01	6.80E-01
**Histidine**	1.121	1.019	0.863	8.88	17.55	8.71	8.14E-02	5.33E-02	3.33E-01	2.65E-01	1.39E-01	6.48E-01
**Isobutyrate**	1.651	1.105	1.168	-37.59	-65.21	-29.42	6.82E-02	4.63E-02	3.86E-01	2.56E-01	1.27E-01	6.48E-01
**Isoleucine**	1.003	0.810	1.112	10.28	25.64	15.46	1.19E-01	1.15E-01	3.99E-01	2.94E-01	1.87E-01	6.48E-01
**Lactate**	0.872	0.853	0.968	19.75	18.67	-1.09	9.45E-02	6.79E-03	7.21E-01	2.79E-01	3.21E-02	7.81E-01
**Leucine**	0.787	0.876	1.128	6.31	17.32	11.04	2.23E-01	9.36E-02	3.69E-01	4.84E-01	1.68E-01	6.48E-01
**Lysine**	0.723	0.981	1.432	0.79	21.44	20.66	6.41E-01	2.96E-02	4.25E-02	7.57E-01	9.05E-02	4.67E-01
**Lysine + Leucine**	0.723	0.939	1.091	-0.40	13.15	13.55	9.76E-01	1.78E-01	3.69E-01	9.76E-01	2.64E-01	6.48E-01
**NAC (2.092 ppm) (s)**	0.787	0.691	1.110	1.86	4.27	2.41	4.34E-01	2.95E-01	6.26E-01	6.27E-01	3.93E-01	7.40E-01
**O-Phosphocholine**	0.856	1.193	0.559	-5.28	3.26	8.53	5.42E-01	6.80E-02	5.82E-01	7.05E-01	1.46E-01	7.29E-01
**Phenylealanine**	0.683	0.715	1.005	2.92	15.07	12.16	6.73E-01	1.04E-01	3.42E-01	7.57E-01	1.74E-01	6.48E-01
**Proline**	0.981	0.907	1.144	14.74	19.33	4.62	7.36E-02	1.53E-01	7.90E-01	2.56E-01	2.34E-01	8.38E-01
**Propylene glycol**	1.234	1.135	0.844	22.41	45.86	24.07	1.22E-01	4.08E-04	1.18E-01	2.94E-01	7.07E-03	4.77E-01
**Pyruvate**	1.105	0.993	0.964	58.44	40.04	-19.54	5.94E-02	9.06E-02	6.80E-01	2.56E-01	1.68E-01	7.68E-01
**sn-G3P**	0.680	0.942	0.615	5.34	12.90	7.57	1.24E-01	9.67E-02	3.93E-01	2.94E-01	1.68E-01	6.48E-01
**Succinate + 3HB**	0.535	1.076	0.505	-1.76	-6.28	-4.52	8.65E-01	3.65E-01	6.56E-01	8.82E-01	4.74E-01	7.58E-01
**Taurine**	1.107	1.019	0.732	10.95	21.15	10.26	2.15E-02	2.76E-02	2.43E-01	1.33E-01	8.96E-02	5.68E-01
**Tryptophan**	0.711	1.160	1.381	10.44	31.09	20.82	2.57E-01	7.22E-04	1.00E-01	5.05E-01	8.57E-03	4.77E-01
**Tyrosine**	1.153	1.304	0.968	14.32	30.04	15.89	2.06E-02	2.25E-03	2.56E-01	1.33E-01	1.53E-02	5.68E-01
**Unk 4 (1.074 ppm) (s)**	1.121	0.571	0.846	2.39	10.37	7.98	5.59E-01	5.15E-01	6.17E-01	7.09E-01	5.90E-01	7.40E-01
**Unk 6 (1.23 ppm) (m)**	0.807	0.814	0.728	-7.81	1.98	9.78	6.21E-01	5.22E-01	5.72E-01	7.51E-01	5.90E-01	7.29E-01
**Unk_10 (2.769 ppm) (s)**	1.505	1.125	0.882	-66.95	-38.54	30.36	2.31E-02	6.26E-02	1.13E-01	1.33E-01	1.42E-01	4.77E-01

**Unk_11 (2.945 ppm) (s)**	0.832	1.149	1.377	-3.69	22.31	25.95	8.45E-01	7.01E-02	1.18E-02	8.79E-01	1.46E-01	2.11E-01
**Unk_12 (3.051 ppm) (s)**	0.409	0.884	0.415	11.65	34.56	23.14	4.84E-01	1.25E-02	1.38E-01	6.81E-01	5.41E-02	5.11E-01
**Unk_13 (3.112 ppm) (s)**	0.341	0.859	0.713	13.17	49.32	36.75	5.31E-01	2.47E-01	2.73E-01	7.05E-01	3.48E-01	5.68E-01
**Unk_14 (3.246 ppm) (s)**	0.568	0.631	0.646	-2.53	1.73	4.26	5.90E-01	8.60E-01	7.04E-01	7.30E-01	8.94E-01	7.79E-01
**Unk_15 (3.63 ppm) (s)**	0.738	0.965	1.301	4.24	19.31	15.11	3.23E-01	5.75E-02	1.22E-02	5.60E-01	1.42E-01	2.11E-01
**Unk_16 (7.334 ppm) (t)**	0.720	1.021	1.098	1.79	17.02	15.24	8.03E-01	2.36E-02	1.75E-01	8.52E-01	8.18E-02	5.68E-01
**Unk_18 (8.238 ppm) (s)**	0.744	0.593	0.770	15.94	2.18	-13.77	3.36E-01	6.84E-01	4.16E-01	5.64E-01	7.28E-01	6.56E-01
**Unk_2 (0.914 ppm) (d)**	1.443	1.082	0.515	-30.80	-28.14	2.72	4.41E-03	1.33E-01	5.89E-01	5.73E-02	2.09E-01	7.29E-01
**Unk_3 (0.967 ppm) (s)**	0.808	0.770	0.885	1.82	16.96	15.15	6.84E-01	3.85E-02	2.41E-01	7.57E-01	1.11E-01	5.68E-01
**Unk_7 (1.444 ppm) (d)**	1.172	0.866	1.237	23.78	25.44	1.69	1.11E-02	1.16E-03	9.35E-01	1.15E-01	1.00E-02	9.72E-01
**Unk_8 (2.050 ppm) (m)**	0.802	1.047	1.559	-2.77	-5.94	-3.17	6.74E-01	6.86E-01	4.93E-01	7.57E-01	7.28E-01	6.80E-01
**Unk_9 (2.743 ppm) (s)**	1.259	0.923	0.796	-21.82	-20.18	1.66	9.65E-02	1.88E-01	5.00E-01	2.79E-01	2.72E-01	6.80E-01
**Valine**	0.941	0.953	1.130	8.63	23.34	14.79	2.87E-02	1.86E-02	1.51E-01	1.36E-01	6.89E-02	5.22E-01

Grey – Metabolites above VIP 1.00, p value <0.05, adjusted p value <0.05

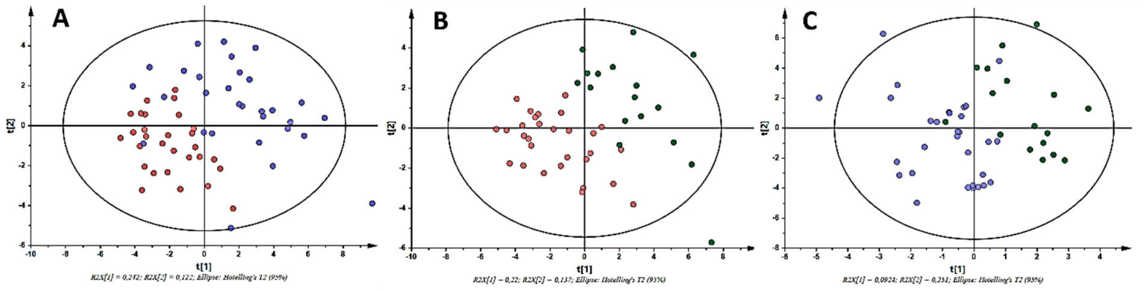

Figure S2. PLS-DA model plots for AS treatment at various time points. A – before treatment vs. after 3 months, B – before treatment vs. after 6 months, C – after 3 months vs. after 6 months of therapy with TNFi. Red – AS patients before treatment, Blue – 3 months after initialization of anti-TNF treatment, Green – 6 months after treatment with TNFi.

Table S3. The VIP score for discrimination PLS with univariate analysis results for quantified metabolites in comparisons for AS patients.

**Ankylosing spondylitis**												
**Metabolite**	**VIP score for PLS-DA model**			**Precentage diffrence [%]**			***p* value**			**Adjusted *p* value**		
	**BT vs 3M**	**BT vs 6M**	**3M vs 6M**	**BT vs 3M**	**BT vs 6M**	**3M vs 6M**	**BT vs 3M**	**BT vs 6M**	**3M vs 6M**	**BT vs 3M**	**BT vs 6M**	**3M vs 6M**
**2-Hydroxybutyrate**	0.752	0.728	0.478	-1.32	-7.26	-5.94	8.39E-01	2.18E-01	6.79E-01	8.77E-01	3.00E-01	9.85E-01
**2-Oxoisocaproate**	1.210	1.291	0.783	32.06	38.28	6.42	1.19E-05	1.22E-02	4.28E-01	1.54E-04	5.76E-02	9.64E-01
**3-Hydroxybutyrate**	0.448	0.748	0.429	-2.41	-4.51	-2.10	6.46E-01	6.89E-01	9.52E-01	7.31E-01	7.46E-01	9.85E-01
**3-Methyl-2-oxovalerate**	1.125	1.151	0.330	36.66	35.26	-1.45	5.76E-04	3.99E-02	9.85E-01	2.99E-03	9.82E-02	9.85E-01
**Acetate**	0.469	0.935	1.859	-2.64	-20.17	-17.55	5.37E-01	1.12E-02	3.52E-02	6.35E-01	5.76E-02	3.17E-01
**Acetone**	1.532	0.902	0.995	-12.87	-9.73	3.14	2.24E-02	1.90E-01	5.10E-01	4.66E-02	2.82E-01	9.82E-01
**Alanine**	0.952	0.927	0.309	15.89	14.42	-1.48	1.11E-04	2.42E-02	9.80E-01	8.22E-04	7.48E-02	9.85E-01

**Choline**	0.915	0.565	1.898	-17.00	9.85	26.74	8.22E-02	1.60E-01	9.33E-02	1.26E-01	2.52E-01	4.86E-01
**Citrate**	1.460	0.858	1.406	36.08	19.67	-16.71	8.55E-07	3.39E-02	8.25E-02	4.44E-05	9.26E-02	4.86E-01
**Creatine**	1.018	1.094	0.592	19.54	26.35	6.90	2.41E-05	8.02E-03	3.56E-01	2.39E-04	5.76E-02	9.64E-01
**Creatinine**	0.895	0.859	0.516	11.87	11.62	-0.26	5.44E-04	2.82E-02	9.64E-01	2.99E-03	8.16E-02	9.85E-01
**Ethanol**	2.288	2.496	0.381	-31.22	-30.42	0.82	4.73E-06	1.00E-04	9.73E-01	8.19E-05	4.12E-03	9.85E-01
**Formate**	1.166	0.969	0.267	28.30	24.75	-3.62	8.69E-03	1.32E-01	3.61E-01	2.38E-02	2.21E-01	9.64E-01
**Glucose**	0.488	0.946	0.583	1.24	1.89	0.66	6.78E-01	9.09E-01	7.78E-01	7.50E-01	9.27E-01	9.85E-01
**Glutamate**	0.678	0.818	0.701	3.03	6.69	3.66	4.64E-01	6.62E-01	4.43E-01	5.74E-01	7.36E-01	9.64E-01
**Glutamine**	1.247	0.961	0.932	17.87	12.55	-5.35	1.78E-06	1.89E-01	4.21E-02	4.62E-05	2.82E-01	3.17E-01
**Glycerol**	0.495	0.375	0.392	8.55	4.28	-4.27	1.83E-02	4.74E-01	3.83E-01	3.96E-02	5.74E-01	9.64E-01
**Glycine**	0.845	0.510	0.707	11.61	6.02	-5.60	5.78E-03	2.16E-01	2.85E-01	1.77E-02	3.00E-01	9.27E-01
**Histidine**	1.054	1.094	0.153	16.77	15.59	-1.18	2.76E-05	1.39E-02	9.68E-01	2.39E-04	5.91E-02	9.85E-01
**Isobutyrate**	0.795	1.223	2.901	-10.88	-89.48	-80.56	4.23E-01	7.39E-04	1.00E-04	5.36E-01	7.97E-03	5.20E-03
**Isoleucine**	0.855	0.859	0.827	13.07	4.47	-8.61	4.75E-02	5.37E-01	5.04E-01	7.96E-02	6.21E-01	9.82E-01
**Lactate**	0.895	1.301	1.101	16.88	31.58	14.91	5.32E-03	1.11E-02	2.51E-01	1.73E-02	5.76E-02	8.70E-01
**Leucine**	1.012	0.997	0.592	12.85	13.49	0.65	1.15E-02	1.19E-02	6.86E-01	2.86E-02	5.76E-02	9.85E-01
**Lysine**	0.788	0.740	0.723	10.85	7.35	-3.50	3.46E-02	1.39E-01	8.17E-01	6.20E-02	2.26E-01	9.85E-01
**Lysine + Leucine**	0.822	0.792	0.693	5.21	3.13	-2.07	2.70E-01	4.40E-01	9.17E-01	3.70E-01	5.72E-01	9.85E-01
**NAC (2.092 ppm) (s)**	1.045	0.941	0.539	0.79	0.63	-0.16	6.21E-01	8.74E-01	9.67E-01	7.17E-01	9.09E-01	9.85E-01
**O-Phosphocholine**	0.607	0.603	0.701	-0.71	6.52	7.22	9.58E-01	9.69E-01	5.88E-01	9.58E-01	9.69E-01	9.85E-01
**Phenylealanine**	0.868	1.269	1.393	11.61	20.89	9.34	5.26E-02	7.66E-04	1.44E-01	8.54E-02	7.97E-03	6.25E-01
**Proline**	1.032	0.805	0.550	10.95	9.42	-1.54	2.78E-02	2.19E-01	9.31E-01	5.15E-02	3.00E-01	9.85E-01
**Propylene glycol**	1.177	1.183	1.489	29.49	51.72	23.11	1.03E-02	4.94E-02	1.73E-01	2.67E-02	1.10E-01	6.91E-01
**Pyruvate**	1.045	1.166	0.909	40.83	75.20	37.23	9.40E-02	5.08E-02	4.55E-01	1.40E-01	1.10E-01	9.64E-01
**sn-G3P**	1.414	1.115	1.788	21.17	7.39	-13.83	5.20E-04	2.51E-01	1.11E-02	2.99E-03	3.35E-01	2.89E-01
**Succinate + 3HB**	0.856	0.806	0.750	11.32	5.75	-5.58	6.95E-02	4.86E-01	3.40E-01	1.10E-01	5.75E-01	9.64E-01
**Taurine**	0.921	0.870	0.465	9.71	14.75	5.06	2.58E-02	8.89E-02	6.10E-01	4.97E-02	1.65E-01	9.85E-01
**Tryptophan**	1.082	1.107	0.363	20.16	23.81	3.69	2.95E-03	1.55E-02	5.94E-01	1.02E-02	5.91E-02	9.85E-01
**Tyrosine**	0.825	1.135	0.511	13.63	17.82	4.22	6.76E-03	1.59E-02	5.84E-01	1.95E-02	5.91E-02	9.85E-01
**Valine**	0.958	0.977	0.591	12.39	12.64	0.25	1.43E-02	4.15E-02	6.89E-01	3.24E-02	9.82E-02	9.85E-01
**Unk 4 (1.074 ppm) (s)**	0.666	0.684	0.668	7.11	-0.82	-7.93	5.12E-01	7.36E-01	7.24E-01	6.19E-01	7.81E-01	9.85E-01
**Unk 6 (1.23 ppm) (m)**	0.653	0.634	1.088	0.16	15.43	15.27	9.53E-01	9.87E-02	1.04E-01	9.58E-01	1.71E-01	4.94E-01
**Unk_10 (2.769 ppm) (s)**	0.881	1.052	1.525	-55.25	-86.99	-36.07	1.40E-02	2.16E-03	2.66E-02	3.24E-02	1.87E-02	3.17E-01
**Unk_11 (2.945 ppm) (s)**	1.134	1.165	0.538	29.84	27.28	-2.61	9.09E-04	1.59E-04	5.91E-01	3.84E-03	4.12E-03	9.85E-01
**Unk_12 (3.051 ppm) (s)**	0.659	0.806	0.165	20.81	24.36	3.59	7.07E-04	2.42E-02	4.64E-01	3.34E-03	7.48E-02	9.64E-01
**Unk_13 (3.112 ppm) (s)**	0.748	0.645	0.290	31.63	30.01	-1.66	1.67E-01	4.68E-01	9.22E-01	2.34E-01	5.74E-01	9.85E-01
**Unk_14 (3.246 ppm) (s)**	0.692	1.042	1.814	4.01	-13.19	-17.18	3.65E-01	5.28E-02	3.71E-02	4.75E-01	1.10E-01	3.17E-01
**Unk_15 (3.63 ppm) (s)**	1.232	0.974	0.934	14.22	9.43	-4.80	9.61E-04	2.44E-02	2.24E-01	3.84E-03	7.48E-02	8.31E-01
**Unk_16 (7.334 ppm) (t)**	0.737	0.780	0.923	5.14	8.41	3.27	2.80E-01	6.71E-02	4.33E-01	3.73E-01	1.29E-01	9.64E-01
**Unk_18 (8.238 ppm) (s)**	0.359	0.340	0.546	0.59	10.12	9.54	8.43E-01	4.57E-01	9.09E-01	8.77E-01	5.74E-01	9.85E-01
**Unk_2 (0.914 ppm) (d)**	1.174	1.345	1.479	-28.50	-56.70	-29.39	2.57E-03	3.50E-04	9.34E-02	9.56E-03	6.06E-03	4.86E-01
**Unk_3 (0.967 ppm) (s)**	0.743	0.659	0.683	8.32	5.19	-3.14	1.60E-01	6.65E-01	9.19E-01	2.30E-01	7.36E-01	9.85E-01
**Unk_7 (1.444 ppm) (d)**	1.090	1.032	0.324	15.65	18.41	2.78	4.46E-02	6.49E-02	6.64E-01	7.73E-02	1.29E-01	9.85E-01

**Unk_8 (2.050 ppm) (m)**	1.619	1.039	0.713	-12.17	-10.84	1.34	2.33E-02	9.78E-02	7.84E-01	4.66E-02	1.71E-01	9.85E-01
**Unk_9 (2.743 ppm) (s)**	0.620	1.051	1.466	3.43	-19.68	-23.07	7.47E-01	3.65E-02	4.27E-02	8.09E-01	9.48E-02	3.17E-01

Grey – Metabolites above VIP 1.00, *p* value <0.05, adjusted *p* value <0.05

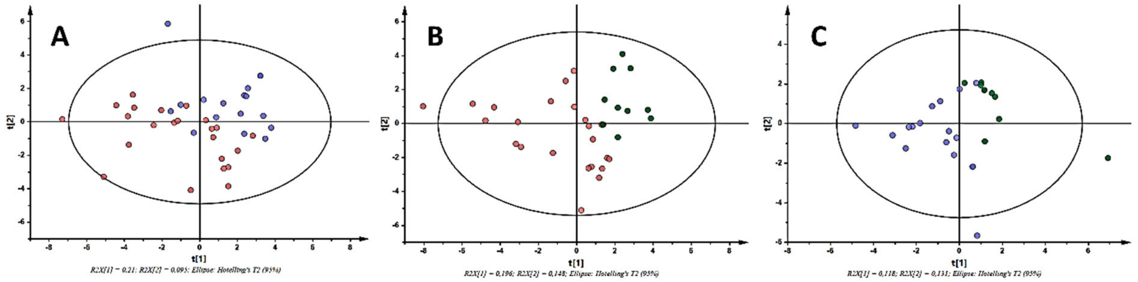

Figure S3. PLS-DA model plots for PsA treatment at various time points. A – before treatment vs. after 3 months, B – before treatment vs. after 6 months, C – after 3 months vs. after 6 months of therapy with TNFi. Red – PsA patients before treatment, Blue – 3 months after initialization of anti-TNF treatment, Green – 6 months after treatment with TNFi.

Table S4. The VIP score for discrimination PLS with univariate analysis results for quantified metabolites in comparisons for PsA group.

**Metabolite**	**VIP score for PLS-DA model**			**Precentage diffrence [%]**			***p* value**			***Adjusted p* value**		
	**BT vs 3M**	**BT vs 6M**	**3M vs 6M**	**BT vs 3M**	**BT vs 6M**	**3M vs 6M**	**BT vs 3M**	**BT vs 6M**	**3M vs 6M**	**BT vs 3M**	**BT vs 6M**	**3M vs 6M**
**2-Hydroxybutyrate**	0.903	0.744	0.452	-19.13	-24.93	-5.87	3.07E-01	2.43E-01	7.64E-01	7.25E-01	5.41E-01	9.26E-01
**2-Oxoisocaproate**	0.145	0.892	1.384	-3.59	19.97	23.52	4.48E-01	1.49E-01	6.37E-02	8.31E-01	4.83E-01	6.62E-01
**3-Hydroxybutyrate**	0.458	0.365	0.322	-33.14	-14.14	19.23	9.96E-01	9.15E-01	6.75E-01	9.96E-01	9.66E-01	9.26E-01
**3-Methyl-2-oxovalerate**	0.362	0.427	0.653	7.67	14.14	6.49	8.21E-01	6.26E-01	6.03E-01	9.27E-01	8.26E-01	9.26E-01
**Acetate**	0.833	1.619	1.613	-13.75	-44.14	-30.85	1.55E-02	2.70E-04	3.65E-02	1.61E-01	7.49E-03	6.32E-01
**Acetone**	1.016	1.664	0.899	-9.74	-17.44	-7.73	1.04E-01	5.80E-03	4.03E-01	4.50E-01	6.03E-02	9.15E-01
**Alanine**	1.042	0.668	0.537	-4.83	0.36	5.19	6.43E-01	5.83E-01	4.66E-01	9.07E-01	8.19E-01	9.15E-01
**Choline**	0.857	1.051	0.595	-6.32	-11.49	-5.18	3.97E-01	2.00E-01	6.14E-01	8.25E-01	5.32E-01	9.26E-01
**Citrate**	0.553	1.437	1.453	-6.36	27.87	34.08	4.96E-01	1.38E-01	1.44E-01	8.46E-01	4.78E-01	8.12E-01
**Creatine**	0.515	0.800	1.250	-1.84	-14.43	-12.60	9.63E-01	2.01E-01	4.05E-01	9.82E-01	5.32E-01	9.15E-01
**Creatinine**	1.152	0.901	0.379	1.71	3.43	1.72	5.04E-01	3.49E-01	7.10E-01	8.46E-01	6.25E-01	9.26E-01
**Ethanol**	3.287	2.410	0.444	-50.49	-48.67	1.94	1.00E-04	9.27E-04	7.86E-01	5.20E-03	1.61E-02	9.26E-01
**Formate**	0.380	1.178	2.162	-3.31	-11.76	-8.46	2.43E-01	3.92E-01	4.75E-01	6.32E-01	6.57E-01	9.15E-01
**Glucose**	1.027	0.197	1.430	-16.53	3.70	20.20	1.93E-01	5.01E-01	1.43E-01	6.30E-01	7.23E-01	8.12E-01
**Glutamate**	1.059	0.672	0.731	-19.32	-9.67	9.69	3.50E-02	2.45E-01	3.68E-01	2.40E-01	5.41E-01	9.15E-01
**Glutamine**	0.591	0.670	1.174	-0.78	9.11	9.89	4.32E-01	4.51E-01	2.45E-01	8.31E-01	6.90E-01	9.15E-01
**Glycerol**	1.770	0.860	0.904	-19.54	-12.12	7.47	1.19E-02	2.15E-01	4.29E-01	1.55E-01	5.32E-01	9.15E-01
**Glycine**	0.676	0.136	0.645	-5.57	-0.40	5.17	2.36E-01	8.90E-01	3.29E-01	6.32E-01	9.66E-01	9.15E-01
**Histidine**	0.882	1.256	0.925	-2.70	3.33	6.03	5.56E-01	7.70E-01	2.57E-01	9.04E-01	9.48E-01	9.15E-01
**Isobutyrate**	1.064	1.272	1.700	-2.21	-61.23	-59.22	7.08E-02	1.67E-02	1.14E-03	3.35E-01	1.24E-01	5.93E-02

**Isoleucine**	0.941	0.924	1.167	-0.76	-15.92	-15.16	8.69E-01	3.66E-02	3.00E-01	9.27E-01	1.90E-01	9.15E-01
**Lactate**	0.959	0.094	0.630	2.41	0.11	-2.29	6.86E-01	6.51E-01	7.64E-01	9.07E-01	8.26E-01	9.26E-01
**Leucine**	0.981	1.015	0.533	-4.54	-4.67	-0.13	7.29E-01	7.91E-01	9.95E-01	9.25E-01	9.48E-01	9.95E-01
**Lysine**	1.106	1.084	0.547	-7.41	-12.76	-5.37	2.18E-01	7.08E-02	5.81E-01	6.30E-01	3.07E-01	9.26E-01
**Lysine + Leucine**	1.130	1.085	0.481	-11.00	-12.79	-1.79	1.57E-01	1.14E-01	7.94E-01	6.26E-01	4.36E-01	9.26E-01
**NAC (2.092 ppm) (s)**	1.306	1.283	0.538	-6.05	-6.74	-0.69	3.19E-02	2.12E-02	9.75E-01	2.40E-01	1.38E-01	9.94E-01
**O-Phosphocholine**	0.738	0.809	0.581	-2.13	-10.04	-7.92	6.90E-01	2.12E-01	4.09E-01	9.07E-01	5.32E-01	9.15E-01
**Phenylealanine**	0.845	0.586	1.141	-8.48	1.23	9.71	2.09E-01	8.02E-01	2.60E-01	6.30E-01	9.48E-01	9.15E-01
**Proline**	1.119	0.450	1.164	-15.81	-3.20	12.62	4.15E-02	9.39E-01	1.56E-01	2.40E-01	9.66E-01	8.12E-01
**Propylene glycol**	0.427	0.743	1.501	-8.78	29.98	38.51	3.79E-01	3.44E-01	1.26E-01	8.20E-01	6.25E-01	8.12E-01
**Pyruvate**	0.953	0.413	1.009	32.05	15.20	-17.06	4.96E-01	6.06E-01	9.45E-01	8.46E-01	8.26E-01	9.94E-01
**sn-G3P**	0.418	0.458	0.454	0.69	2.66	1.97	7.60E-01	4.90E-01	5.56E-01	9.27E-01	7.23E-01	9.26E-01
**Succinate + 3HB**	0.636	0.392	0.451	-10.98	-7.71	3.27	8.72E-01	9.47E-01	7.45E-01	9.27E-01	9.66E-01	9.26E-01
**Taurine**	0.456	0.251	0.599	-4.89	-2.41	2.48	2.83E-01	8.48E-01	6.31E-01	7.00E-01	9.66E-01	9.26E-01
**Tryptophan**	0.502	0.599	0.941	-2.32	2.07	4.39	6.77E-01	9.42E-01	3.42E-01	9.07E-01	9.66E-01	9.15E-01
**Tyrosine**	1.026	1.122	0.859	2.19	11.42	9.24	7.85E-01	3.27E-01	3.38E-01	9.27E-01	6.25E-01	9.15E-01
**Valine**	1.094	1.013	0.393	1.71	1.77	0.07	6.98E-01	3.46E-01	9.30E-01	9.07E-01	6.25E-01	9.94E-01
**Unk 4 (1.074 ppm) (s)**	0.910	0.963	0.679	-1.00	13.80	14.79	8.14E-01	4.44E-01	5.08E-01	9.27E-01	6.90E-01	9.26E-01
**Unk 6 (1.23 ppm) (m)**	1.045	0.944	0.821	-8.02	-20.20	-12.23	6.45E-01	1.65E-01	4.60E-01	9.07E-01	5.03E-01	9.15E-01
**Unk_10 (2.769 ppm) (s)**	0.772	1.246	1.463	-20.16	-88.36	-71.38	1.86E-01	3.20E-02	5.63E-02	6.30E-01	1.85E-01	6.62E-01
**Unk_11 (2.945 ppm) (s)**	0.177	0.150	1.035	-1.75	2.43	4.18	5.98E-01	3.66E-01	5.99E-01	9.07E-01	6.34E-01	9.26E-01
**Unk_12 (3.051 ppm) (s)**	0.464	1.026	1.346	-9.42	49.01	57.76	9.46E-01	2.50E-01	2.21E-01	9.82E-01	5.41E-01	9.15E-01
**Unk_13 (3.112 ppm) (s)**	0.650	0.535	1.389	-36.94	-47.80	-11.36	3.43E-01	4.10E-01	8.70E-01	7.75E-01	6.66E-01	9.63E-01
**Unk_14 (3.246 ppm) (s)**	1.147	1.503	1.474	-16.78	-30.70	-14.11	4.03E-02	1.25E-02	9.08E-02	2.40E-01	1.08E-01	7.87E-01
**Unk_15 (3.63 ppm) (s)**	0.771	0.395	0.891	-1.50	0.40	1.90	8.08E-01	6.46E-01	5.64E-01	9.27E-01	8.26E-01	9.26E-01
**Unk_16 (7.334 ppm) (t)**	1.214	1.051	0.161	-9.82	-8.49	1.33	5.10E-02	2.93E-01	8.70E-01	2.65E-01	6.09E-01	9.63E-01
**Unk_18 (8.238 ppm) (s)**	0.574	1.065	0.666	-7.87	3.63	11.49	5.85E-01	8.79E-01	4.17E-01	9.07E-01	9.66E-01	9.15E-01
**Unk_2 (0.914 ppm) (d)**	1.085	1.618	1.723	-27.34	-68.57	-43.26	3.91E-03	2.88E-04	1.85E-02	6.77E-02	7.49E-03	4.81E-01
**Unk_3 (0.967 ppm) (s)**	0.757	0.932	0.431	-9.86	-14.41	-4.57	4.37E-01	1.17E-01	7.38E-01	8.31E-01	4.36E-01	9.26E-01
**Unk_7 (1.444 ppm) (d)**	0.912	0.289	0.907	-0.76	-6.07	-5.32	8.74E-01	9.72E-01	9.66E-01	9.27E-01	9.72E-01	9.94E-01
**Unk_8 (2.050 ppm) (m)**	1.475	1.673	0.580	-20.35	-24.40	-4.09	2.30E-03	3.31E-03	7.69E-01	5.98E-02	4.30E-02	9.26E-01
**Unk_9 (2.743 ppm) (s)**	0.940	1.067	0.405	-13.92	-21.06	-7.19	1.96E-01	5.14E-02	8.01E-01	6.30E-01	2.43E-01	9.26E-01

Grey – Metabolites above VIP 1.00, *p* value <0.05, adjusted *p* value <0.05

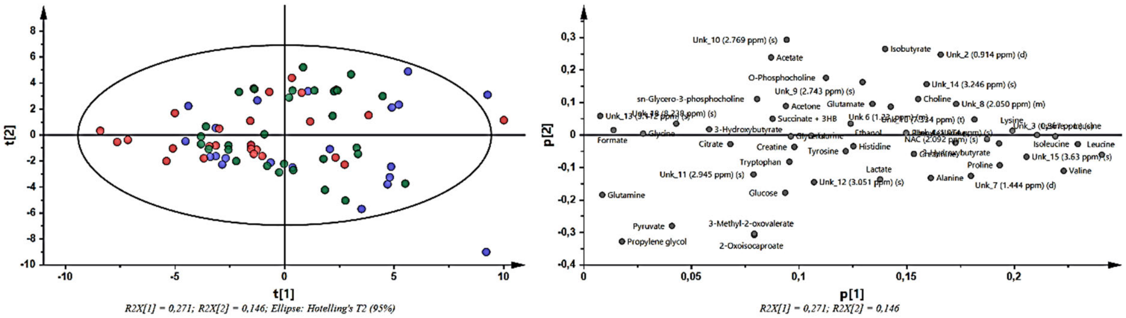

Figure S4. PCA model plot and corresponding loading plot for the RA, AS and PsA groups of patients before treatment. Red – RA before treatment Green – AS before treatment, Blue – PsA before treatment.

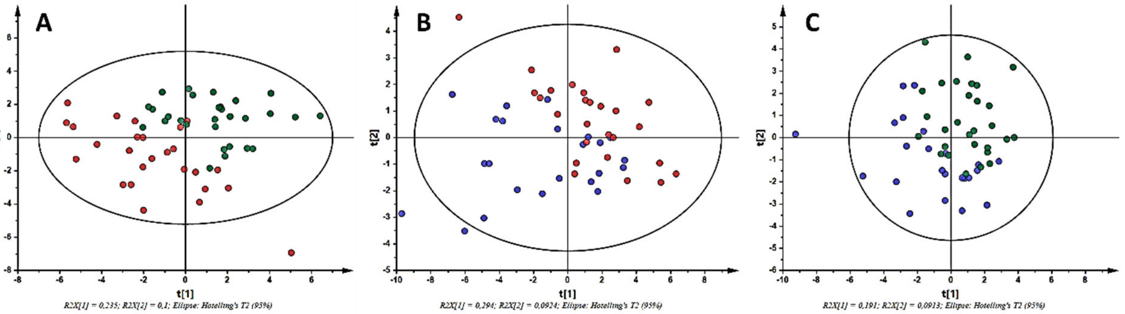

Figure S5. PCA model plot and corresponding loading plot for the RA, AS and PsA groups of patients before treatment. A – RA vs. AS, B – RA vs. PsA, C – AS vs. PsA. Red – RA before treatment Green – AS before treatment, Blue – PsA before treatment.

Figure S6. The MetPA analysis within the most perturbed biochemical pathways in: a) RA, b) AS, c) PsA

a)

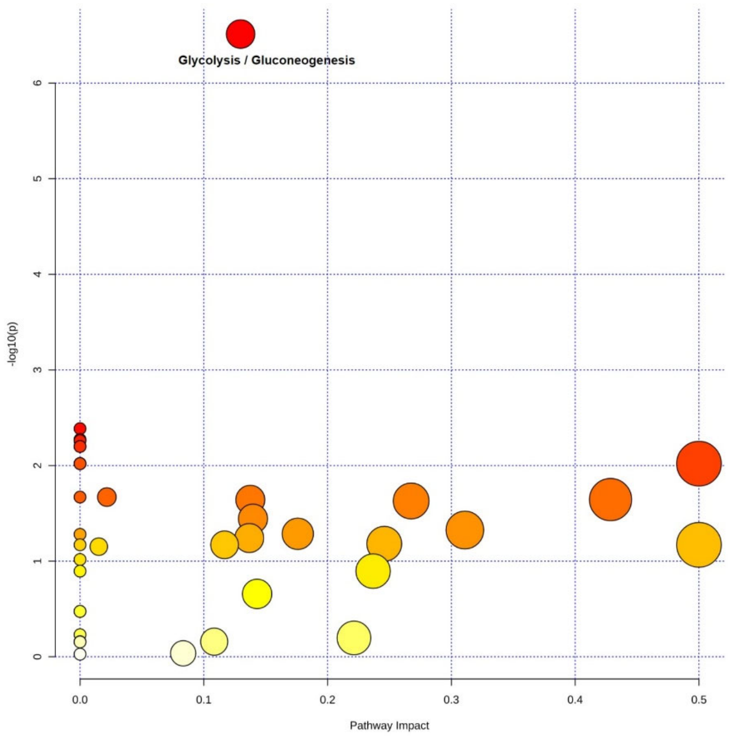

b)

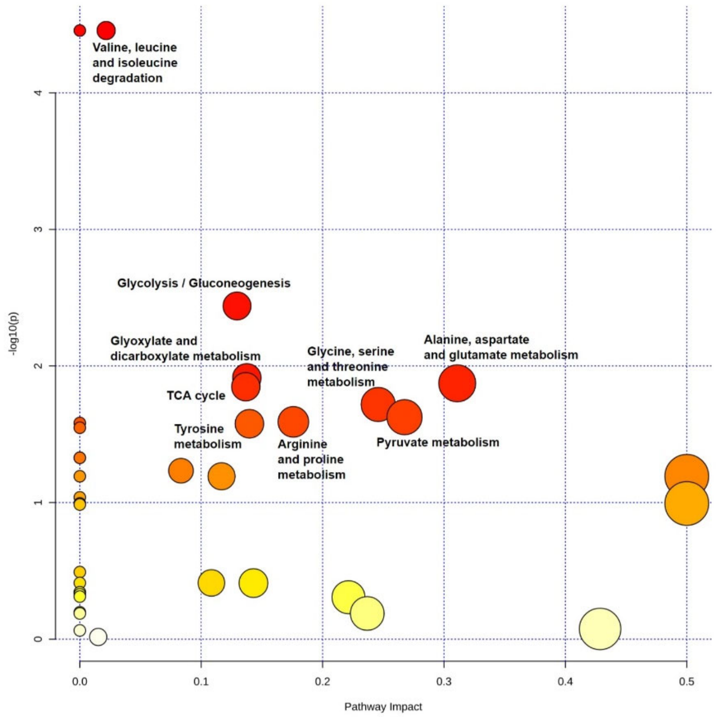

c)

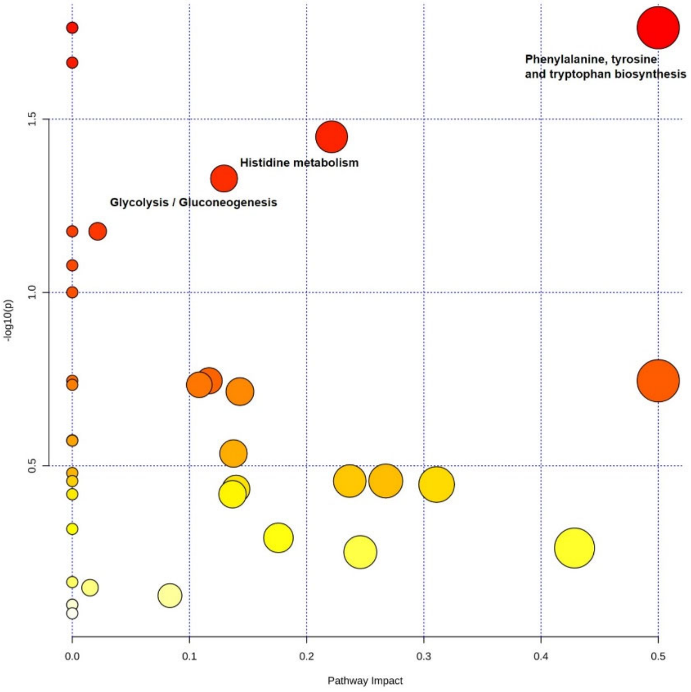

Table S5. List of assignments of ^{1}H NMR resonance signals with chemical shift, KEGG and HMDBI ID. Presented in alphabetical order list of compounds spin systems that were used in multivariate and univariate analysis.

Name	Chemical shift [ppm]	Multiplicity	HMDB ID	KEGG ID
**2-Oxoisocaproate**	0.924; 2.623	(d), (d)	HMDB00695	C00233
**2-Hydroxybutyrate**	0.904	(s)	HMDB00008	C05984
**3-Hydroxybutyrate**	1.207; 2.324	(d), (dd)	HMDB00357	C01089
**3-Methyl-2-oxovalerate**	1.101	(d)	HMDB00491	C03465
**Acetate**	1.932	(s)	HMDB00042	C00033
**Acetone**	2.248	(s)	HMDB01659	C00207
**Alanine**	1.492	(d)	HMDB00161	C00041
**Choline**	3.199	(s)	HMDB00097	C00114
**Citrate**	2.557	(dd)	HMDB00094	C00158
**Creatine**	3.046; 3.942	(s); (s)	HMDB00064	C00300
**Creatinine**	3.061; 4.076	(s); (s)	HMDB00562	C00791
**Ethanol**	1.189	(t)	HMDB00108	C00469
**Formate**	8.464	(s)	HMDB00142	C00058
**Glycine**	3.582	(s)	HMDB00123	C00037
**Glucose**	3.275; 3.522; 3.895; 5.263	(m); (m); (d); (d)	HMDB00122	C00031
**Glutamate**	2.349	(m)	HMDB00148	C00025
**Glutamine**	2.152; 2.45	(m); (m)	HMDB00641	C00064
**Glycerol**	3.588	(m)	HMDB00131	C00116
**Isobutyrate**	1.078	(s)	HMDB01873	C02632
**Isoleucine**	0.998	(d)	HMDB00172	C00407

**Lactate**	1.338; 4.133	(d); (q)	HMDB00190	C00186
**Leucine**	0.951	(t)	HMDB00687	C00123
**Lysine + Leucine**	1.737	(m)	-	-
**Lysine**	1.92; 3.033	(m); (t)	HMDB00182	C00047
**NAC**	2.092	(s)	-	-
**O-Phosphocholine**	3.211	(s)	HMDB00284	C00588
**Histidine**	8.178	(s)	HMDB00177	C00135
**Phenylealanine**	7.437	(m)	HMDB00159	C00079
**Proline**	2.017	(m)	HMDB00162	C00148
**Propylene glycol**	1.128	(d)	HMDB01881	C02912
**Pyruvate**	2.391	(s)	HMDB00243	C00022
**sn-Glycero-3-phosphocholine**	3.235	(s)	HMDB00086	C00670
**Succinate + 3HB**	2.409	(s)	-	-
**Taurine**	3.296	(s)	HMDB00251	C00245
**Tryptophan**	7.562	(d)	HMDB00883	C00183
**Tyrosine**	6.925	(m)	HMDB00158	C00082
**Valine**	0.977	(d)		
**Unk_2**	0.914	(d)	-	-
**Unk_3**	0.967	(s)	-	-
**Unk_4**	1.074	(s)	-	-
**Unk_6**	1.23	(m)	-	-
**Unk_7**	1.444	(d)	-	-
**Unk_8**	2.05	(m)	-	-
**Unk_9**	2.743	(s)	-	-
**Unk_10**	2.769	(s)	-	-
**Unk_11**	2.945	(s)	-	-
**Unk_12**	3.051	(s)	-	-
**Unk_13**	3.112	(s)	-	-
**Unk_14**	3.246	(s)	-	-
**Unk_15**	3.63	(s)	-	-
**Unk_16**	7.334	(t)	-	-
**Unk_18**	8.238	(s)	-	-
